# Treatment of Real-World HCV Genotype 2-Infected Japanese Patients with Sofosbuvir plus Ribavirin

**DOI:** 10.3390/biology6020030

**Published:** 2017-05-09

**Authors:** Tatsuo Kanda, Masato Nakamura, Shin Yasui, Yuki Haga, Akinobu Tawada, Eiichiro Suzuki, Yoshihiko Ooka, Koji Takahashi, Reina Sasaki, Shuang Wu, Shingo Nakamoto, Makoto Arai, Fumio Imazeki, Osamu Yokosuka

**Affiliations:** 1 Department of Gastroenterology and Nephrology, Graduate School of Medicine, Chiba University, 1-8-1 Inohana, Chuo-ku, Chiba 260-8670, Japan; nkmr.chiba@gmail.com (M.N.); ntcph863@yahoo.co.jp (S.Y.); hagayuki@gmail.com (Y.H.); eiichiro0709@hotmail.com (E.S.); ooka-y@umin.ac.jp (Y.O.); koji517@gmail.com (K.T.); reina_sasaki_0925@yahoo.co.jp (R.S.); gosyou100@yahoo.co.jp (S.W.); araim-cib@umin.ac.jp (M.A.); yokosukao@faculty.chiba-u.jp (O.Y.); 2 Safety and Health Organization, Chiba University, 1-33 Yayoicho, Inage-ku, Chiba 263-8522, Japan; akinobutawada@hospital.chiba-u.jp (A.T.); imazekif@faculty.chiba-u.jp (F.I.); 3 Department of Molecular Virology, Graduate School of Medicine, Chiba University, 1-8-1 Inohana, Chuo-ku, Chiba 260-8670, Japan; nakamotoer@yahoo.co.jp

**Keywords:** genotype 2, hepatitis C virus, ribavirin, sofosbuvir, treatment-experienced

## Abstract

The aim of this study was to characterize the treatment response and tolerability of sofosbuvir plus ribavirin therapies in Japanese patients infected with hepatitis C virus (HCV) genotype (GT)-2. This retrospective study analyzed 114 Japanese HCV GT-2 patients treated for 12 weeks with 400 mg of sofosbuvir plus weight-based ribavirin daily. This treatment led to higher sustained virologic response at 12-weeks post-treatment (SVR12) rates in both treatment-naïve and treatment-experienced patients. The efficacy of this treatment in compensated cirrhotics was the same as that in patients with chronic hepatitis. HCV GT-2a infection and lower estimated glomerular filtration rates (eGFR) tended to be associated with SVR12. Of 114 patients, 113 completed the combination of sofosbuvir plus ribavirin for 12 weeks. Seven patients without SVR12 did not have HCV NS5B-S282 mutations. The overall SVR12 rate was 90.4% (103 of 114). More effective therapeutic options with less adverse events are desired to achieve higher SVR rates in HCV GT-2 Japanese patients.

## 1. Introduction

Hepatitis C virus (HCV) infection causes acute and chronic hepatitis, hepatic cirrhosis and hepatocellular carcinoma (HCC) [1]. Chronic HCV infection is one of the major causes of HCC in Japan and the United States [1,2]. Previous studies demonstrated that the eradication of HCV by interferon-including regimens could improve liver-related and non-liver related mortality [3,4]. Interferon-free regimens against HCV infection eradicate this virus with higher sustained virologic response (SVR) rates and less adverse events [5].

There are at least seven HCV genotypes worldwide [6]. HCV genotype (GT)-2 accounts for almost 30% of all HCV infected patients in Japan [4,7]. Previous standard therapy based on a combination of peginterferon plus ribavirin for 12 weeks could lead to ~80% SVR rates in HCV GT-2 infected patients [4,8]. As SVR rates of retreatment with a combination of peginterferon plus ribavirin for HCV GT-2 patients were only 60–70% [9], and these treatments often possessed some severe adverse events, more effective therapeutic options with less adverse events are desired.

Sofosbuvir is an oral nucleotide analogue inhibitor of HCV NS5B polymerase [10]. Ribavirin is an oral synthetic guanosine analogue and has antiviral activities against DNA and RNA viruses [11]. Twelve or 16 weeks of treatment with sofosbuvir and ribavirin resulted in an SVR of 78% in HCV GT-2 or GT-3 patients, for whom interferon treatment was not an option, and of 50 to 73% in GT-2 or GT-3 patients who experienced prior treatment failure [10]. In a Japanese phase 3 study [12], treatment with sofosbuvir plus weight-based ribavirin led to 97% SVR with fewer adverse events in HCV GT-2 patients. The Japanese health insurance system approved this regimen as standard care for the treatment of HCV GT-2 patients in July 2015. However, there have been few reports of real-world studies, and data from this regimen in Japanese patients remains limited.

Rapid virologic response (RVR, HCV RNA negative at week 4 after the commencement of treatment) is one of the good predictors for SVR in the interferon-based treatment for HCV GT-2 patients [13]. In the peginterferon and ribavirin era, we demonstrated that the assessment of serum HCV RNA 24 weeks after end of treatment (EOT) using TaqMan PCR was more relevant than 12 weeks for the prediction of SVR [14]. Approximately 2% of patients who achieved an SVR at 12 weeks after EOT (SVR12) did not achieve an SVR at 24 weeks after EOT (SVR24) [15]. Although SVR12 could not always predict persistent virologic response [16], we used SVR12 as an index for persistent virologic response in the present study because SVR24 could not be available in some patients. In the present retrospective study, our aim is to characterize the treatment response and tolerability of sofosbuvir plus ribavirin therapies in Japanese patients infected with HCV GT-2.

## 2. Patients and Methods

### 2.1. Patients

This single-center cohort consisted of 114 consecutive Japanese patients who commenced 12 week treatments with 400 mg of sofosbuvir daily (Sovaldi, Gilead Sciences, Tokyo, Japan) plus weight-based ribavirin (400–1000 mg daily) (Rebetol, MSD, Osaka, Japan, or Copegas, Chugai Pharma, Tokyo, Japan) between July 2015 and June 2016 at Chiba University Hospital (Table 1). A total of 75 treatment-naïve patients and 39 interferon-treated patients were included. Several cases were previously reported [17,18,19]. Eligible patients were 20 years of age and older and infected with HCV GT-2 at the baseline. Exclusion criteria were as follows: (1) Child-Pugh B or C cirrhosis; (2) severe anemia at the baseline; (3) severe renal dysfunction at the baseline; (4) present existence of HCC; and (5) any serious medical condition of any other organ such as arrhythmia, congestive heart failure, or other hepatic diseases. Patients with a history of curative treatment of HCC were included.

This retrospective study was approved by the Ethics Committee of Chiba University, School of Medicine (number 1462 and 1753). Participation in the study was posted at our institutions. This study conformed to the ethical guidelines of the Declaration of Helsinki.

### 2.2. Clinical and Laboratory Assessments

Clinical parameters were measured by standard laboratory techniques at a central laboratory in Chiba University Hospital. Blood samples were obtained at the baseline, weeks 4, 8 and 12 and then 4, 8 and 12 weeks after the end of treatment. HCV serotyping and genotyping were performed as described previously [20]. HCV RNA was measured by COBAS TaqMan HCV assay version 2.0 (Roch Diagnostics, Tokyo, Japan), with a lower limit of quantification of 15 IU/mL. Rapid virologic response (RVR) was defined as undetectable HCV RNA at week 4 after the commencement of treatment. EOT response (EOTR) was defined as undetectable HCV RNA at the end of treatment. SVR at 4, 8, or 12 weeks (SVR4, SVR8, or SVR12) was also used as an evaluation of virologic response.

The HCV NS5B resistance-associated variant (RAV) at S282 was determined by a commercial direct-sequencing assay (SRL Laboratory, Tokyo, Japan).

Cirrhosis was diagnosed by liver biopsy, transient elastography (Fibroscan of greater than 12 kPa) and/or ultrasonography (sign of cirrhosis). These findings were observed within 1 year before treatment. HCC were excluded by imaging modalities such as ultrasonography, computed tomography (CT), and/or gadolinium ethoxybenzyl diethlenetriamine pentaacetic acid (Gd-EOB-DTPA) enhanced magnetic resonance imaging (MRI).

### 2.3. Statistical Analysis

Data were expressed as the mean ± standard deviation (SD). Statistical analyses were conducted. Univariate analyses were performed using Student’s *t*-test or a Chi-squared test. Variables with *p* < 0.05 at univariate analysis were evaluated using multivariate logistic regression analysis. For all tests, two-sided *p* values were calculated, and the results were considered statistically significant at *p* < 0.05. Statistical analysis was performed using Excel Statistics program for Windows 2010 (SSRI, Tokyo, Japan).

## 3. Results

### 3.1. Characteristics of the Patients in the Present Study

On the basis of the inclusion and exclusion criteria, 114 HCV GT-2 patients were included. Demographic and baseline characteristics classified by past-interferon use are shown in Table 1. Of them, 65.8% (*n* = 75) were treatment-naïve and 34.2% (*n* = 39) have experienced interferon treatment. Of the 39 treatment-experienced patients, 21, 4, 8 and 6 were relapsers of peginterferon plus ribavirin, null responders of peginterferon plus ribavirin, interferon-intolerant patients and non-responders to standard interferon monotherapy, respectively. Ages of the treatment-naïve patients were significantly lower than those of the treatment-experienced patients. Aspartate aminotransferase (AST), alanine aminotransferase (ALT) and estimated glomerular filtration rates (eGFR) levels were significantly lower in the treatment-experienced patients than those in the treatment-naïve patients (Table 1).

### 3.2. Virologic Response

**Figure 1:**
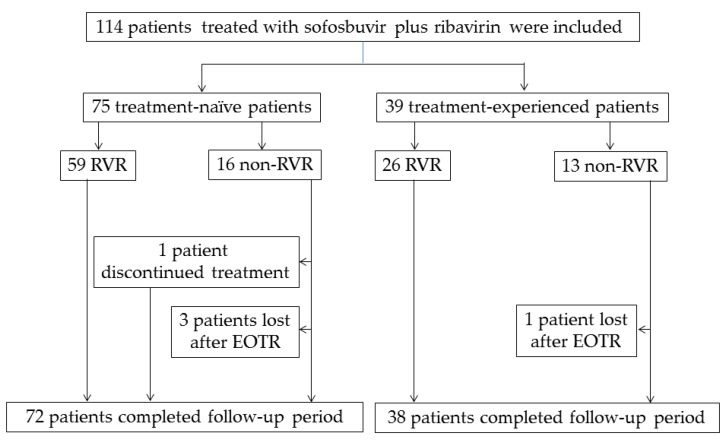
Study profile. Of 114 patients, 113 completed the combination of sofosbuvir plus ribavirin, and only one patient discontinued treatment. RVR, rapid virologic response; EOTR, end of treatment response.

After 4 weeks of treatment with sofosbuvir plus ribavirin, HCV RNA was undetectable in 85 of 114 (74.6%) patients; 59 of 75 (78.6%) and 26 of 39 (66.7%) of the treatment-naïve and treatment-experienced patients achieved RVR, respectively (Figure 1). There were no significant differences in RVR rates between the two groups (*p* = 0.242). In three treatment-naïve patients, HCV RNA was detectable at 8 weeks. Two of the three patients continued treatment and achieved EOTR, SVR4 and SVR 12. Only one patient discontinued sofosbuvir plus ribavirin treatment at 2 weeks due to the patient’s will and exacerbation of depression, and his HCV RNA did not disappear at the end of treatment.

Virologic responses are shown in Table 2. An overall intention-to-treat EOTR was achieved in 113 of 114 (99.1%) patients. SVR4 was achieved in 108 of 114 (94.7%); among treatment-naïve patients, SVR4 was achieved in 71 of 75 (94.6%) patients, and among treatment-experienced patients, SVR4 was achieved in 37 of 39 (94.9%) patients. SVR8 was achieved in 104 of 114 (91.2%) patients; among treatment-naïve patients, SVR8 was achieved in 67 of 75 (89.3%) patients, and among treatment-experienced patients, SVR8 was achieved in 37 of 39 (94.9%) patients. SVR12 was achieved in 103 of 114 (90.4%) patients; among treatment-naïve patients, SVR12 was achieved in 66 of 75 (88.0%), and among treatment-experienced patients, SVR12 was achieved in 37 of 39 (94.9%) patients.

### 3.3. Efficacy for Patients with or without Cirrhosis

**Figure 2:**
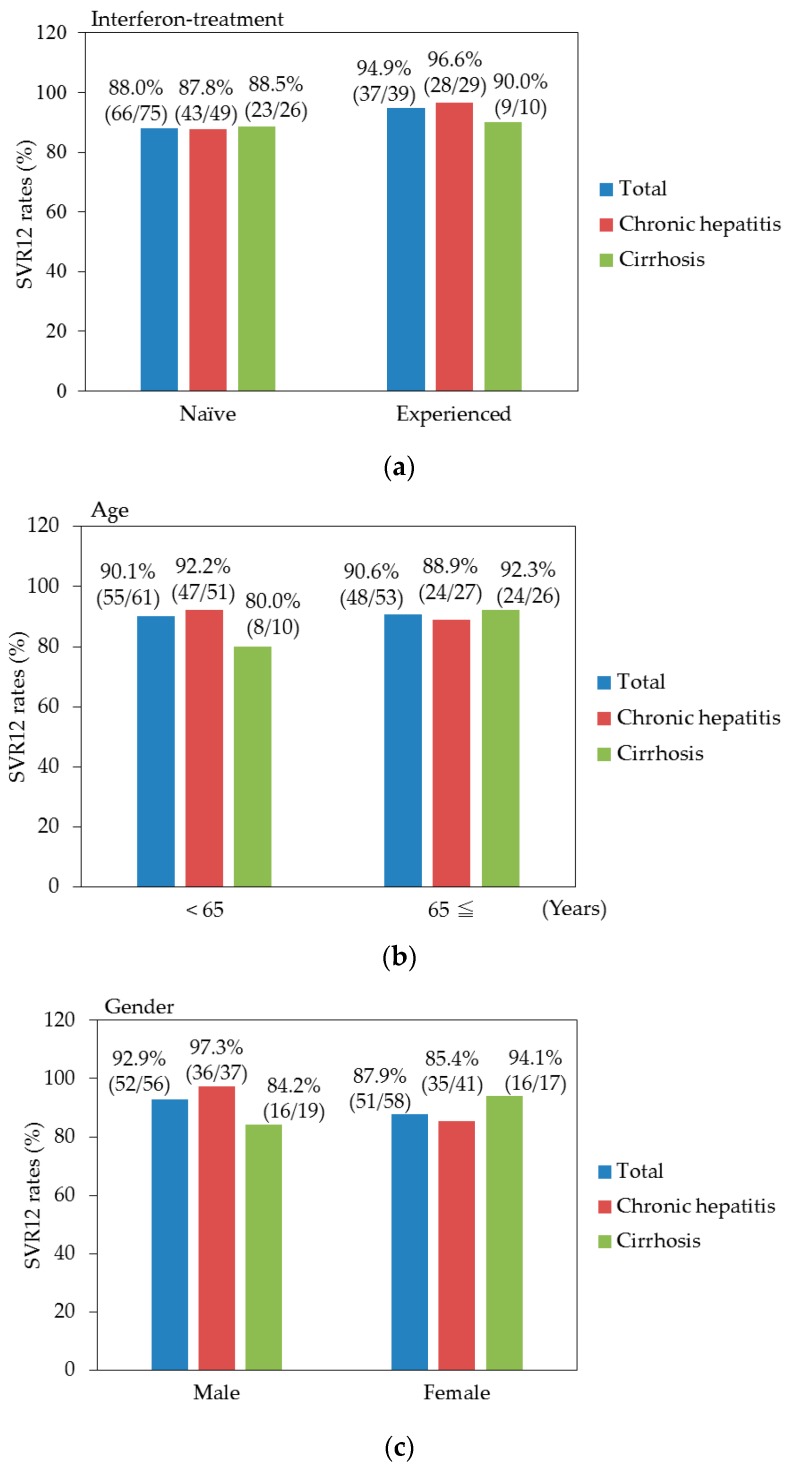

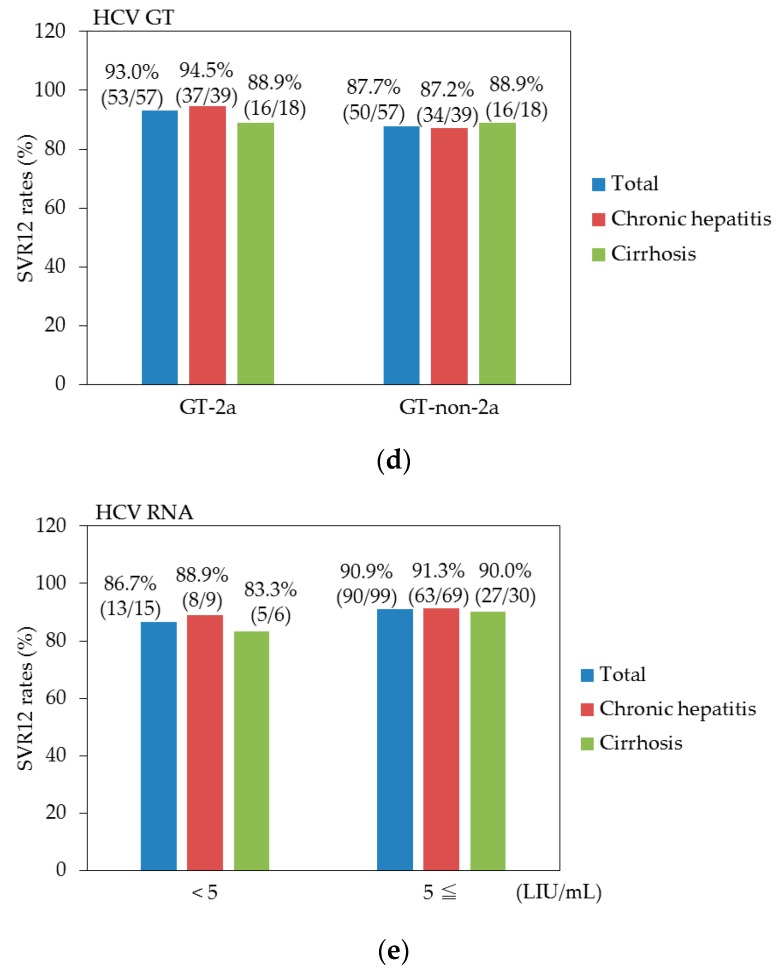
Sustained virologic response at 12 weeks (SVR 12) rates for the various groups with or without cirrhosis. (**a**) Previous interferon treatment; (**b**) Age; (**c**) Gender; (**d**) HCV genotype (GT); and (**e**) HCV RNA viral load. LIU/mL, log IU/mL.

Due to its adverse events, it was difficult to treat patients with cirrhosis by peginterferon plus ribavirin therapy [5]. In the present study, sofosbuvir plus ribavirin treatment could lead to higher SVR12 rates of patients with or without cirrhosis [32/36 (88.9%) and 71/78(91.0%), respectively]. We also analyzed SVR12 rates in various subgroups with or without cirrhosis (Figure 2a–e). The efficacy of sofosbuvir plus ribavirin treatment in compensated cirrhotics was the same as that in patients with chronic hepatitis. We did not find any significant difference among these subgroups. SVR12 rates of sofosbuvir plus ribavirin treatment in compensated cirrhotics were much higher than those previously reported in peginterferon plus ribavirin therapy [5].

### 3.4. Factors at Baseline Associated with SVR

A total of 103 patients for whom HCV RNA could be evaluated 12 weeks after stopping treatment were analyzed (Table 3). Although the number of study patients was limited, univariate analysis showed that HCV GT-2a infection and lower eGFR tended to be associated with SVR12. These factors were also analyzed by multivariate logistic regression analysis. Multivariate analysis showed that HCV GT-2a infection (odds ratio, 6.37; 95% CI, 0.73–55.6; *p* = 0.094) and a lower eGFR (<78 mL/min/1.73 m^2^) (odds ratio, 3.58; 95% CI, 0.73–17.5; *p* = 0.116) tended to be associated with SVR12.

### 3.5. Adherence and Safety

It has been reported that HCV GT-1 patient adherence to peginterferon plus ribavirin therapy is advantageous for the treatment response [21]. Of 114 patients, 113 completed the combination treatment of sofosbuvir plus ribavirin for 12 weeks, and only one patient discontinued treatment due to the patient’s will and his worsening depression. Of note, anemia due to ribavirin was relatively well controlled and did not cause the discontinuation of therapy in the present study. As a severe adverse event, one patient drowned in the bath and died 4 weeks after stopping the treatment. Table 4 shows that seven patients failed the sofosbuvir plus ribavirin treatment. Breakthrough during treatment was not observed. Three, two and one patients relapsed at 4, 8 and 12 weeks, respectively, after the cessation of the treatment. These seven patients did not have HCV NS5B-S282 mutations, which were associated with virologic failure of sofosbuvir [22,23], and did not use any proton pump inhibitors (PPIs) [24]. One problem was that follow-ups were lost in four patients, in whom eradication of HCV could not be confirmed.

## 4. Discussion

There have been few reports on the efficacy and safety of sofosbuvir plus ribavirin treatment in real-world Japanese patients infected with HCV GT-2 [17,18,19,25,26]. In the present study, we analyzed 114 HCV GT-2 patients treated with sofosbuvir plus ribavirin treatment in Chiba University Hospital, one of the urban hospitals near Tokyo in Japan. The SVR12 rates of treatment-naïve and treatment-experienced patients were 66/75 (88.0%) and 37/39 (94.9%), respectively (Table 2). Japanese phase 3 trials reported that 88/90 (97.8%) of treatment-naïve patients achieved SVR12, and 60/63 (95.2%) of treatment-experienced patients achieved SVR12 [12]. The SVR12 rates in the present study were lower than those in [12]. This may be because the number of participants in our study was small, and four patients were lost in the follow up, although Chang et al. [27] reported that SVR12 was also particularly low among patients with HCV GT-2 (80%) who were treated with sofosbuvir plus ribavirin for 12 weeks in a cohort of Asian American patients. They included patients with decompensated cirrhosis, which may be the reason why the lower SVR rates were found in that study [27].

Of note, 113 of 114 patients completed the combination of sofosbuvir plus ribavirin for 12 weeks. The inosine triphosphatase (ITPA) polymorphism may influence hemoglobin levels and incidence of ribavirin dose reduction during sofosbuvir plus ribavirin therapy [26]. Although we did not measure the ITPA polymorphism in the present study as previously studies have [28], it was reported that the ITPA polymorphism did not appear to correlate with clinical outcome [24,29,30], and we do not have to measure the ITPA polymorphism before the 12-week treatment of sofosbuvir plus ribavirin. Moreover, the adherence of these drugs for 12 weeks appeared much better than the previous standard of care against HCV GT-2 with peginterferon plus ribavirin for 24 weeks [8,9].

A previous study on peginterferon plus ribavirin showed that interleukin-28B (IL28B) polymorphism is useful predictor of SVR [28]. In the present study, the IL28B polymorphism was examined in only 30 patients; 15 of 16 patients with the IL28B major genotype achieved SVR and one patient with the IL28B major genotype was lost; eight of nine patients with the IL28B minor genotype achieved SVR and one patient with the IL28B minor genotype did not achieve SVR. Further studies on the IL28B polymorphism will be needed.

We found that HCV GT-2a infection and lower eGFR (<78 mL/min/1.73 m^2^) tended to be independent predictors for SVR12 (Table 3). Elfiky et al. [31] reported that sofosbuvir is a better direct-acting antiviral agent for HCV GT-1a and GT-3b than for GT-2b and GT-4a. HCV NS5A inhibitor ledipasvir plus sofosbuvir for 12 weeks may be more effective for the treatment of HCV GT-2 and led to 96% SVR rates (25/26) [32]. This regimen shows promise for being effective for HCV GT-2 and avoids the use of ribavirin. As the number of the study was small [28], further studies should be needed. The SVR rate in the sofosbuvir-NS5A inhibitor velpatasvir group was 99% among patients with HCV GT-2 [33]. Twelve weeks of treatment with these regimens results in SVR rates that were superior to those with sofosbuvir plus ribavirin in HCV GT-2 patients [32,33]. These treatments could be alternative therapeutic options for HCV GT-2 patients. Of interest, lower eGFR (<78 mL/min/1.73 m^2^) tended to be associated with SVR12, although sofosbuvir may be used safely down to an eGFR of 30 mL/min/1.73 m^2^ [34].

SVR12 rates of patients with history of HCC were 7/8 (87.5%). Differences in SVR12 within this group were not found to be statistically significant, although this might be due to limited statistical power due to a small sample size. It has been reported that HCC recurrence rates were much higher and occurred much earlier than would be expected [35] and that scientific and clinical evidence for immune-related recurrence of HCC after interferon-free treatment of HCV call for caution [36].

One hepatitis B virus (HBV)-infected patient with HBV surface antigen-positive and two HIV-infected patients were included in the present study. Although there were several reports of HBV reactivation during interferon-free treatment [37,38], attention should be paid to HBV-infected patients. Sofosbuvir plus ribavirin seems to be a useful treatment option for patients co-infected with HIV and HCV GT-2 [39].

## 5. Conclusions

We found that sofosbuvir plus ribavirin for 12 weeks could lead to higher SVR rates with less adverse events in Japanese real-life patients infected with HCV GT-2 and that 94.9% SVR rates are achieved in interferon-treatment experienced patients with HCV GT-2. We also observed that HCV GT-2a infection and lower eGFR (<78 mL/min/1.73 m^2^) tended to be associated with SVR12 in HCV GT-2-infected patients treated with sofosbuvir plus ribavirin. Therapeutic options other than sofosbuvir plus ribavirin are needed for the much higher SVR rates in HCV GT-2 patients.

## Figures and Tables

**Table 1:** Characteristics of 114 hepatitis C virus (HCV) genotype (GT)-2 patients at the commencement of treatment.

Characteristics	All (*n* = 114)	Treatment-Naïve (*n* = 75)	Treatment-Experienced (*n* = 39)	*p*-Values ^1^
Age (years)	62.0 ± 12.5	60.2 ± 13.3	65.6 ± 10.0	0.028
Gender (male/female)	56/58	38/37	18/21	0.795
Interferon (naive/experienced)	75/39	75/0	0/39	
HCV GT (2a/2b/unknown)	57/46/11	39/31/5	18/15/6	0.920
HCV RNA (L/H)	15/99	11/64	4/35	0.712
Body weight (kg)	61.9 ± 12.1	61.8 ± 12.7	62.2 ± 11.2	0.869
Body length (cm)	162.3 ± 8.8	163.2 ± 8.8	160.4 ± 8.7	0.108
Histories of HCC +/−	8/106	5/70	3/36	0.855
Chronic hepatitis/cirrhosis	78/36	49/26	29/10	0.441
Liver stiffness (kPa)	10.2 ± 8.3	10.5 ± 8.7	9.6 ± 7.5	0.584
AST (IU/L)	50.6 ± 38.7	56.1 ± 43.8	38.8 ± 20.7	0.022
ALT (IU/L)	54.5 ± 51.6	62.8 ± 58.4	36.9 ± 25.7	0.010
Hemoglobin (g/dL)	13.5 ± 1.4	13.6 ± 1.5	13.3 ± 1.3	0.291
Platelets (×10^4^/μL)	16.5 ± 6.1	16.8 ± 6.7	15.8 ± 4.7	0.407
eGFR (mL/min/1.73 m^2^)	78.0 ± 20.2	80.7 ± 20.5	72.2 ± 18.6	0.032

Data are expressed as the mean ± SD. HCV RNA: L, <5.0 LIU/mL and H, ≥5.0 LIU/mL; AST, aspartate aminotransferase; ALT, alanine aminotransferase; eGFR, estimated glomerular filtration rates. ^1^ *p*-values, treatment-naïve versus treatment-experienced groups.

**Table 2:** Response during and after treatment.

Characteristics	All (*n* = 114)	Treatment-Naïve (*n* = 75)	Treatment-Experienced (*n* = 39)	*p*-Values ^1^
*HCV undetectable no. (%)*				
*During treatment*				
At 4 w	85 (74.6)	59 (78.7)	26 (66.7)	0.242
At 8 w	111 (97.4)	72 (96.0)	39 (100)	0.516
At 12 w	113 (99.1)	74 (98.7)	39 (100)	0.738
*After treatment*				
Post 4 w	108 (94.7)	72 (96.0)	37 (94.9)	0.839
Post 8 w	104 (91.2)	67 (89.3)	37 (94.9)	0.520
Post 12 w	103 (90.4)	66 (88.0)	37 (94.9)	0.398
Virologic failure				
Discontinuation	1	1/75 (1.3)	0/39 (0)	0.738
Relapse	6	5/72 (6.9)	1/38 (2.6)	0.613

^1^ *p*-values, treatment-naïve versus treatment-experienced groups.

**Table 3:** Univariate and multivariate logistic regression analysis of predictors of SVR12.

Characteristics	SVR (*n* = 103)	Non-SVR (*n* = 7)	Univariate	Multivariate
			*p*-values ^1^	*p*-values ^1^
Age (years)	62.4 ± 12.4	61.6 ± 13.7	0.870	
Gender (male/female)	52/51	2/5	0.464	
Interferon (naive/experienced)	66/37	6/1	0.451	
HCV GT (2a/others)	53/50	1/6	0.131	0.094
HCV RNA (L/H)	13/90	1/6	0.647	
Body weight (kg)	61.7 ± 12.2	63.4 ± 17.2	0.729	
Body length (cm)	162.7 ± 8.8	159.5 ± 9.9	0.357	
Histories of HCC +/−	7/96	1/7	0.913	
Chronic hepatitis/cirrhosis	71/32	5/2	0.776	
Liver stiffness (kPa)	10.2 ± 8.3	8.4 ± 3.1	0.571	
AST (IU/L)	50.6 ± 38.7	48.0 ± 29.2	0.862	
ALT (IU/L)	54.5 ± 51.6	48.3 ± 29.9	0.754	
Hemoglobin (g/dL)	13.5 ± 1.4	13.3 ± 1.3	0.714	
Platelets (×10^4^/μL)	16.5 ± 6.1	17.0 ± 9.8	0.841	
eGFR (mL/min/1.73 m^2^)	70.5 ± 17.5	88.4 ± 26.7	0.0129	0.116

Data are expressed as the mean ± SD. SVR, sustained virologic response; HCV RNA: L, <5.0 LIU/mL and H, ≥5.0 LIU/mL; AST, aspartate aminotransferase; ALT, alanine aminotransferase; eGFR, estimated glomerular filtration rates. ^1^ *p*-values, SVR versus non-SVR groups.

**Table 4:** Seven patients who failed to respond to sofosbuvir plus ribavirin treatment.

No.	Age/Gender	Treatment	GT	Cirrhosis	Efficacies	Adherence >80%	NS5B-S282	Others
1	62/Male	Naive	2b	Yes	Discontinued at 2 w	No	Wild	
2	43/Female	Naive	2b	No	Relapse (post 4 w)	Yes	Wild	
3	63/Female	Naive	2a	No	Relapse (post 4 w)	Yes	Wild	
4	74/Female	Naive	2	No	Relapse (post 8 w)	No ^1^	Wild	
5	79/Male	Naive	2b	Yes ^1^	Relapse (post 8 w)	Yes	Wild	SR-BII ^2^
6	44/Female	Naive	2b	No	Relapse (post 12 w)	Yes	Wild	
7	66/Female	Experienced	2b	No	Relapse (post 4 w)	Yes	Wild	

^1^ Post-curative treatment of hepatocellular carcinoma; ^2^ SR-BII, stomach remnant after Billroth II operation.
